# Editorial: Advanced Nanomaterials for Light-Emitting Diodes and Solar Cells

**DOI:** 10.3389/fchem.2021.741760

**Published:** 2021-07-29

**Authors:** Baiquan Liu, Qifan Xue, Xuyong Yang, Swee Tiam Tan

**Affiliations:** ^1^ School of Electronics and Information Technology, Sun Yat-sen University, Guangzhou, China; ^2^ Institute of Polymer Optoelectronic Materials and Devices, State Key Laboratory of Luminescent Materials and Devices, South China University of Technology, Guangzhou, China; ^3^ Key Laboratory of Advanced Display and System Applications of Ministry of Education, Shanghai University, Shanghai, China; ^4^ School of Energy and Chemical Engineering, Xiamen University Malaysia, Sepang, Malaysia

**Keywords:** nanomaterial, optoelectronic, interface, morphology, mechanism

Thanks to the outstanding optoelectronic properties, advanced nanomaterials have received increasing attention from both academia and industry (Liu et al., 2020). In recent years, intensive effort has been devoted to developing high-performance nanomaterials, which enables huge potential in wide optoelectronic applications (Kong et al., 2021; Niu et al., 2021), particularly for light-emitting diodes (LEDs) and solar cells (SCs). It is our great pleasure to introduce this Special Issue entitled “Advanced Nanomaterials for Light-Emitting Diodes and Solar Cells”. This Special Issue highlights the major significance of material-device research from different perspectives, combining both the modern experimental approaches and theoretical simulations. We present a collection of 10 featured articles from this exciting field that covers the emerging concepts, strategies and techniques of advanced nanomaterials for the development of LEDs and SCs.

Simplified organic LED (OLED) structure with feasible fabrication process plays a critical role in illumination. Xu et al. incorporated ultrathin non-doped emissive nanolayers (0.3 nm), demonstrating low efficiency roll-off and simple-structure OLEDs. Meanwhile, Xie et al. developed solution-processed blue thermally activated delayed fluorescence OLEDs with a narrow full-width at half-maximum of 32 nm by employing a molecule containing boron and nitrogen atoms as guest emitter, obtaining high color purity OLEDs. On the other hand, it is important to develop novel solution-processed hole injection materials for high-performance OLEDs. Zhu et al. synthesized the molybdenum disulfide quantum dots (MoS_2_ QDs) and demonstrated green phosphorescent OLEDs with hybrid poly (3,4-ethylenedioxythiophene)/poly (styrenesulfonate) (PEDOT:PSS)/QDs hole injection layer. The OLED with PEDOT:PSS/MoS_2_ hole injection layer exhibited a maximum current efficiency of 72.7 cd A^−1^, which is 28.2% higher than that of the OLEDs with single PEDOT:PSS, indicating an effective way to achieve the high efficiency OLEDs with sulfide QDs as the hole injection layer.

GaN-based LED is also a promising device for lighting and displays. Zhang et al. systematically investigated the effect of mesa size-reduction in InGaN/GaN LEDs in two lateral dimensions both experimentally and numerically, providing insights on device miniaturization. While Lu et al. fabricated and demonstrated strain-reduced micro-LEDs in various sizes and investigated the size effect on the optical properties and the indium concentration for the quantum wells. Their work provides the rules of thumb to achieve high power performance for micro-LEDs. On the other hand, Liu et al. prepared 2-inch free-standing GaN substrates with a thickness of ∼250 μm on double-polished sapphire substrates by employing a combined buffer layer prepared by hydride vapor phase epitaxy and the laser lift-off technique, giving a route for high-power GaN-based devices.

Global warming and climate change has motivated the community to search for practical sustainable energy sources to replace fossil fuels. SCs provide an effective approach to supply clean and inexhaustible energy. Yu et al. introduced a perylene diimide derivative (PDINO) in organic SCs, passivating the defects between tin oxide (SnO_2_) electron transporting layer (ETL) and the active layer. The power conversion efficiency of the PDINO-modified device was 14.9%, demonstrating a strategy of utilizing the organic-inorganic hybrid ETL to realize high-performance SCs. Besides, the thermoelectric effect that enable direct conversion between thermal and electrical energy was incorporated with SCs via heat management and light harvesting. Wang et al. reviewed the interfacial chemistry and electrical feature of various polymer-inorganic thermoelectric hybrid nanomaterials, and discussed the prospect and challenges of polymer-inorganic nanocomposites in the field of thermoelectric energy.

The investigation on luminescence materials and metal nanomaterials is an important complementary field to LEDs and SCs. Xiao et al. investigated the effect of substitution of BaF_2_ for BaO to improve spectroscopic properties in Tm^3+^ doped gallium tellurite glasses for efficient 2.0 μm fiber laser, indicating that Tm^3+^ doped gallium tellurite glasses containing BaF_2_ was an excellent host material. On the other side, gold nanomaterials can be used not only in optoelectronic fields but also the clinical arena in humans. Jin et al. reviewed the biomedical applications and molecular mechanism of gold nanomaterials in bone and cartilage tissue engineering, and presented the current challenges and future directions.

We would like to take this opportunity to thank all those who have contributed to this Special Issue and all referees for their insightful advice. In addition, we would like to thank *Frontiers in Chemistry* for their excellent editorial support. We hope that this Special Issue will intrigue the researchers worldwide and that future efforts will contribute to the nanomaterials research community.
